# Diversity and determinants of bitterness, astringency, and fat content in cultivated Nacional and native Amazonian cocoa accessions from Ecuador

**DOI:** 10.1002/tpg2.20218

**Published:** 2022-09-06

**Authors:** Kelly Colonges, Edward Seguine, Alejandra Saltos, Fabrice Davrieux, Jérôme Minier, Juan‐Carlos Jimenez, Marie‐Christine Lahon, Darío Calderon, Cristian Subia, Ignacio Sotomayor, Fabián Fernández, Olivier Fouet, Bénédicte Rhoné, Xavier Argout, Marc Lebrun, Pierre Costet, Claire Lanaud, Renaud Boulanger, Rey Gastón Loor Solorzano

**Affiliations:** ^1^ CIRAD UMR AGAP Institut Montpellier F‐34398 France; ^2^ AGAP Institut, Univ. Montpellier, CIRAD, INRAE Institut Agro Montpellier France; ^3^ CIRAD UMR Qualisud Montpellier F‐34398 France; ^4^ Qualisud Univ. Montpellier, Avignon Université, CIRAD, Institut Agro, IRD, Université de La Réunion Montpellier France; ^5^ Seguine Cacao/Guittard Chocolate Co Arroyo Grande CA USA; ^6^ Instituto Nacional de Investigacion Agropecurias, INIAP Quito Ecuador; ^7^ Cirad UMR Qualisud Réunion F‐97400 France; ^8^ Valrhona Tain l'hermitage France

## Abstract

Cocoa (*Theobroma cacao* L.) is the only tree that can produce cocoa. Cocoa beans are highly sought after by chocolate makers to produce chocolate. Cocoa can be fine aromatic, characterized by floral and fruity notes, or it can be described as standard cocoa with a more pronounced cocoa aroma and bitterness. In this study, the genetic and biochemical determinants of sensorial notes and nonvolatile compounds related to bitterness, astringency, fat content, and protein content will be investigated in two populations: a cultivated modern Nacional population and a population of cocoa accessions collected recently in the Ecuadorian South Amazonia area of origin of the Nacional ancestral variety. For this purpose, a genome‐wide association study (GWAS) was carried out on both populations, with results of biochemical compounds evaluated by near‐infrared spectroscopy (NIRS) assays and with sensory evaluations. Twenty areas of associations were detected for sensorial data especially bitterness and astringency. Fifty‐three areas of associations were detected linked to nonvolatile compounds. A total of 81 candidate genes could be identified in the areas of the association.

## Core Ideas

- Two populations of cocoa trees from Ecuador were used for GWAS.
- Variable presence of nonvolatile compounds could be partly explained by genetic variability.
- Candidate genes that may explain genetic variation in bitterness and astringency were identified.

**Abbreviations**
GBS: genotyping‐by‐sequencing
GWAS: genome‐wide association study
LD: linkage disequilibrium
MLM: mixed linear model
NIRS: near‐infrared spectroscopy
SNP: single‐nucleotide polymorphism

## 1 INTRODUCTION

Cocoa (*Theobroma cacao* L.) tree belongs to the Malvaceae family (Bayer & Kubitzki, 2003). It is a tree of great agronomic and economic interest, as it is the only source to produce chocolate. Worldwide consumption of chocolate is constantly increasing and is studied for its health benefits (Yeh et al., 2016).

Cocoa is a diploid plant (2*n* = 2*x* = 20) with a relatively small genome (430 Mb for the Criollo genome) equivalent in size to that of rice (*Oryza sativa* L.) (Argout et al., 2011; IRGSP & Sasaki, 2005; Lanaud et al., 1992). The cocoa genome of two cultivars has been fully sequenced and serves as reference for genomic data analysis: a Criollo cultivar (Argout et al., 2011, 2017) and an Amelonado cultivar (Motamayor et al., 2013). Cocoa shows great genetic diversity. Currently, 10 genetic groups have been identified in cocoa species through genetic analysis (Motamayor et al., 2008). Cacao can be classified into two types of products: bulk cocoa, which has a strong cocoa taste, and aromatic fine cocoa, which is characterized by floral and fruity notes (Sukha et al., 2008). The most widely grown cultivars of fine aromatic cocoa are Nacional, Criollo, and Trinitario. Trinitario cultivars are hybrids between Criollo and Amelonado. The Amelonado is a population variety that produces mostly bulk cocoa. The Criollo cultivar, on the other hand, produces cocoa beans with a predominantly fruity aroma (Lachenaud & Motamayor, 2017). The Criollo cultivar is not widely cultivated because of its low vigor and increased susceptibility to disease (Cheesman, 1944).

The Nacional ancestral variety originated from Ecuador where the currently cultivated cacao trees belong to the modern Nacional cultivars. This hybrid population is the result of several generations of crosses between the ancestral Nacional and Trinitario type trees introduced in Ecuador in the last century (Bartley, 2005; Loor et al., 2009). Surveys were undertaken in the presumed domestication center of Nacional to search for native cocoa trees related to the ancestral Nacional variety (Loor Solorzano et al., 2012, 2015) to enlarge the genetic resources for fine cocoa breeding.

The fine (floral and fruity) flavors of modern Nacional have started to be studied for their volatile compound composition (Cevallos‐Cevallos et al., 2018; Colonges et al., 2021a, 2021b; Luna et al., 2002; Rottiers et al., 2019; Ziegleder, 1990). Nacional cocoa, like all cocoa, also contains nonvolatile compounds, such as polyphenols, caffeine, or theobromine (Wollgast & Anklam, 2000; Zheng et al., 2004), which are known to provide bitterness and astringency to cocoa products (Lesschaeve & Noble, 2005). A high concentration of these compounds can therefore mask the fine flavors of cocoa. However, these compounds do not only bring defects. Thanks to its richness in polyphenols, cocoa contributes to good mental health and cardiovascular protection (Andújar et al., 2012; Tuenter et al., 2018).

To study the genetic and biochemical determinants of bitterness and astringency of the Ecuadorian cocoa trees, as well as their fat and protein contents, two important factors interacting with flavors, the nonvolatile compounds contained in fermented roasted and nonroasted beans, were characterized by NIRS. Sensorial analyses on liquors were also carried out. All these data were used to conduct a genome‐wide association study (GWAS) on all these traits using molecular genotyping data obtained by genotyping‐by‐sequencing (GBS).

### 1.1 MATERIALS AND METHODS

#### 1.1.1 Vegetal material

Two populations of cocoa trees were used for this study. The first population is a population of 169 cocoa trees belonging to the modern Nacional cultivars as previously described (Colonges et al., 2021a, 2021b). In this document, this population is referred to as the ‘Nacional population’.

The second population used is composed of 202 cocoa trees. They belong to surveys carried out in the domestication center of the ancestral Nacional variety in Ecuador previously identified (Loor Solorzano et al., 2012, 2015). The collected trees were put into a germplasm collection located at an agricultural college in Pangui and in two Instituto Nacional de Investigacion Agropecurias experimental centers in Pichilingue (EET‐P) and in Domono (Supplemental Table S1). In this document, this population is referred to as the ‘Amazonian population’.

**Core Ideas**

- Two populations of cocoa trees from Ecuador were used for GWAS.
- Variable presence of nonvolatile compounds could be partly explained by the genetic variability.
- Candidate genes that may explain genetic variation in bitterness and astringency were identified.

### 1.2 Microfermentation

In both cases, the pods were harvested at maturity in the different growing locations. The microfermentations took place at Pichilingue for the Nacional population and at Domono for the Amazonian population within 24 h of harvest. In both cases, the microfermentations were carried out under the most homogeneous conditions possible. The cocoa beans of each genotype were placed in linen bag nets. They were then distributed over four floors in the middle of the mass of Nacional modern cocoa beans. At 24 and 72 h of fermentation, turning was performed as follows: at each turning step, the bags of beans at the bottom were placed at the top and those in the middle‐low position were placed in the middle‐high and vice versa. After 4.5 d, the beans were taken out of the net and dried separately for each genotype in a greenhouse. When the moisture content was less than or equal to 8%, the beans were considered dry and were placed under vacuum.

### 1.3 Sensorial analysis

For the modern Nacional population, 144 individuals were characterized by sensory analysis based on blind tastings carried out on three replicates per sample. For the Amazonian population, 159 genotypes were characterized. The tastings were conducted on cocoa liquor. The cocoa liquor corresponds to merchantable cocoa (dried fermented beans) that has been roasted and ground. The sensory notes (bitterness, astringency, RoastDegree, Cocoa, and TotalAcidity) were judged with a score ranging from 0 (no note detected) to 10 according to the International Standards for the Assessment of Cocoa Quality and Flavour protocol (The Alliance of Bioversity International and CIAT, 2020). We used the average of the three replicates for the GWAS analysis.

### 1.4 Nonvolatile compounds analysis

Near‐infrared spectroscopy acquisitions and processing were carried out according to the protocol of Davrieux et al. (2007). For the modern Nacional population, these acquisitions were done on fermented, dried, and roasted beans, while for the Amazonian population, these acquisitions were done on fermented and dried beans. These acquisitions made it possible to calculate the concentrations of fat content, caffeine, theobromine, procyanidins B2, procyanidins B5, procyanidins C1, epicatechin, and procyanidins total, and to deduce from them the ratio of theobromine to caffeine and the total procyanidin concentration (Álvarez et al., 2012). With the same method, protein content and NH_3_ concentration were determined for the Amazonian population.

### 1.5 DNA extraction and genotyping

DNA was extracted following the protocol of Risterucci et al. (2000) protocol. DNA samples were genotyped by sequencing using Diversity Arrays Technology Sequencing technology (Kilian et al., 2012) and carried out by the DArT company. The resulting raw reads were recovered and processed as follows. The adapter sequences and low‐quality scores extremities (−q 20) were removed from the reads using Cutadapt (v2.10). The reads with a length <20 bases were filtered. The remaining reads were mapped to the V2 sequence of the Criollo reference genome (Argout et al., 2017) using Burrows–Wheeler Aligner v0.7.15 with the MEM algorithm and standard parameters. Single‐nucleotide polymorphism (SNP) calling was performed using HaplotypeCaller in the Genome Analysis Toolkit (GATK v4.1.9.0) and the final SNP set was established using the GATK VariantFiltration tool with stringent criteria (biallelic SNPs only with at least three reads of the alternate allele to be called as an SNP, depth coverage >6 and <40 per accession and a maximum of 10% missing data). The SNP markers with unknown locations were discarded for analysis.

### 1.6 Genetic analysis

#### 1.6.1 Linkage disequilibrium calculation

For the modern Nacional population, linkage disequilibrium (LD) calculations were performed by Loor (2007).

For the Amazonian population, the LD was calculated with Haploview v4.2 (Barrett et al., 2005) following the method of Sardos et al. (2016). The graphical representation of the LD decay was done with the R package ggplot2 following Sardos et al. (2016).

#### 1.6.2 Genome‐wide association study

For the modern Nacional population, we performed a GWAS with SNP markers associated with biochemical (169 accessions × 5,195 markers) and sensory (144 accessions × 5,195 markers) traits using TASSEL 219 v5. For all the traits, we used a mixed linear model (MLM) the detailed information was described in Colonges et al. (2021a).

For the Amazonian population, through GBS, ∼50,000 SNP markers were detected. Markers with missing data or with a frequency of presence of the minor allele <5% were discarded. After these different filters, 5,337 SNP markers were selected. A GWAS was performed on SNP markers associated with biochemical (202 genotypes × 5,337 markers) and sensory (159 accessions × 5337 markers) traits using the TASSEL v5 software.

For all traits, the choice of the MLM was the most relevant.

After comparison of the quantile–quantile plot, two methods were selected:

1. The use of an MLM model with a kinship matrix considered as a random effect, added as covariables to control the false positive rate was chosen for the association analyses of biochemical compounds.
2. The use of an MLM model with a structure matrix, determined by performing a principal component analyses integrated with TASSEL v5 software, considered as a fixed effect, and with a relatedness matrix considered as a random effect added as covariables to control the false positive rate for the association analyses of sensorial data.

In both cases, the relatedness matrix was constructed using the identity‐by‐state pairwise method proposed by Tassel v5. The option of not compressing and re‐evaluating the variance components for each marker was chosen.

The threshold for significance was determined using the R Simple M package based on the Bonferonni correction (Gao et al., 2010, 2008). For the modern Nacional population, the threshold corresponded to a *p* value of 1.79 × 10^−5^.

For the Amazonian population, the threshold corresponded to a *p* value of 1.68 × 10^−5^.

The physical maps with the representation of the association zones were created using SpiderMap (Rami, 2007 unpublished, Spidermap v1.7.1, free software, CIRAD).

For each significantly associated marker we studied the LD of these markers two by two. Markers with a LD with an *R* ^2^ > .2 were grouped in the same association area as suggested by Alqudah et al. (2020). The mean extent of LD of the Amazonian population is ∼1.2 cM (600 kb) (Colonges et al., 2022). This 600‐kb limit was used to determine the confidence interval of associations. For each positive marker, we report an association zone of ±300 kb, that is, an association zone of 600 kb. If two or more markers have overlapping confidence intervals, they are grouped into a single association zone. The lowest and highest position of the grouped markers represents the confidence intervals of this zone.

Candidate gene identification was performed within the 600‐kb region surrounding using the *T. cacao* genome sequence annotation (Argout et al., 2017).

#### 1.6.3 Statistical analysis

Principal component analyses were performed with the R package Mixomics, and the graphical representations were performed with the R package factoextra. Box plots were performed with the R package ggplot2. Student's *t* tests to check the significance of the differences in the box plots were carried out using the R package stats.

#### 1.6.4 Phylogenetic tree

The phylogenetic tree was generated using DARwin software (Perrier & Jacquemoud‐Collet, 2006). The genetic distances were calculated using the Dice coefficient and the neighbor‐joining method (Dice, 1945; Saitou & Nei, 1987).

## 2 RESULTS

### 2.1 Characterization of biochemicals and sensorial traits in the Nacional population

**FIGURE 1:**
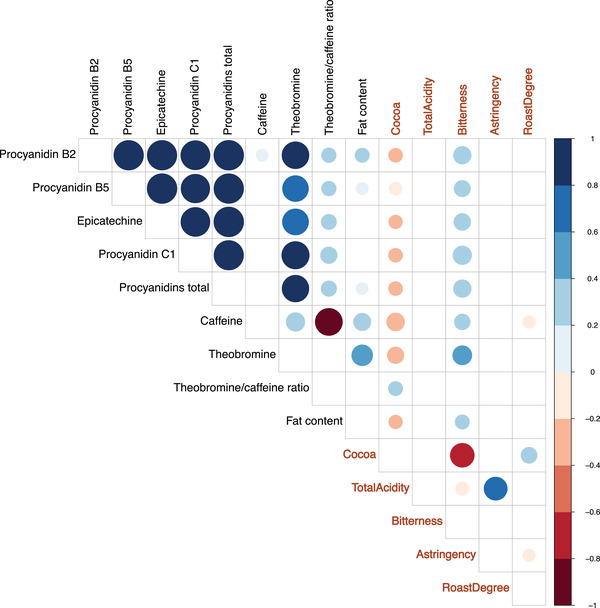
Correlation matrix of the results of the determination of nonvolatile compounds by near‐infrared spectroscopy (in roasted beans) and sensory analysis (in liquors) from cocoa belonging to the modern Nacional population. Nonvolatile compounds are shown in black and sensory traits are shown in bold and brown. The correlations were calculated by the Pearson method. The white boxes represent no significant correlations. The color of the circles corresponds to Pearson's correlation coefficient (R^2^ correlation coefficient). The scale on the right indicates the interpretations of different colors (blue for positive correlation and red for negative correlation). The size of the circles corresponds to the *p* value corresponding to the calculation of each correlation coefficient. The *p* value threshold for a significant correlation is .05

The NIRS analyses revealed the nine biochemical contents for each tree of this population. Strong positive correlations could be observed for these different traits. The presence of all types of detected polyphenols appears to be positively correlated between them (Figure 1). No strong correlation could be observed between the different compounds identified by NIRS and the results of the sensory analyses for astringency and bitterness (Figure 1).

**FIGURE 2:**
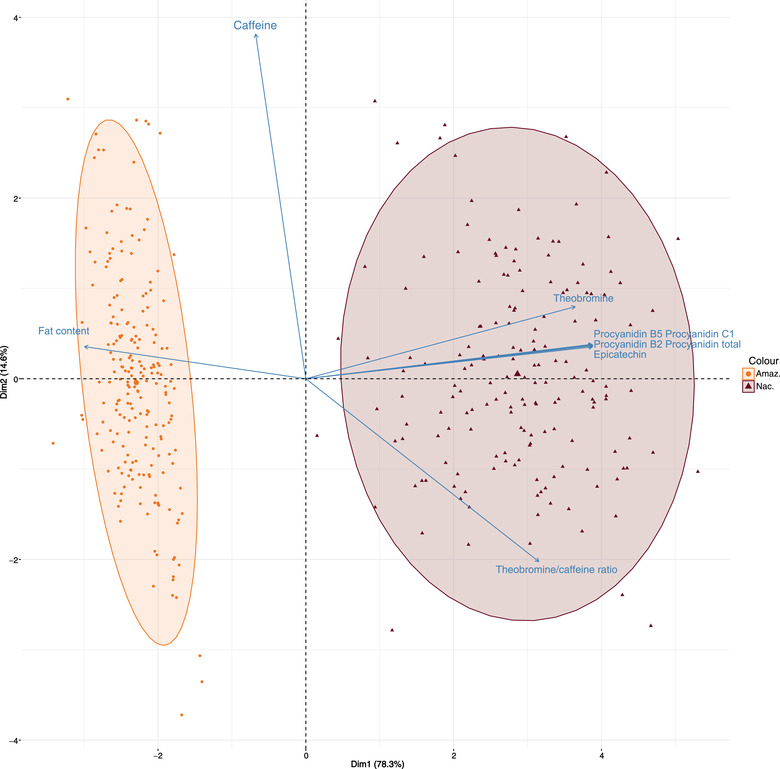
Graphical representation of principal component analyses results. Results corresponding to near‐infrared spectroscopy determinations performed on cocoa beans from the modern Nacional population (in brown) and on cocoa beans from the Amazonian native cocoa tree population (in orange)

Principal component analysis results from the NIRS assays gathering all the traits studied show a continuous variation within the modern Nacional population (Figure 2A). Axis 1 is mainly influenced by the concentrations of total procyanidin, procyanidin B2, and epicatechin. Axis 2 is mostly influenced by the amounts of caffeine, theobromine/caffeine ratio, and fat content.

### 2.2 Characterization of biochemicals and sensorial traits in the Amazonian population

**FIGURE 3:**
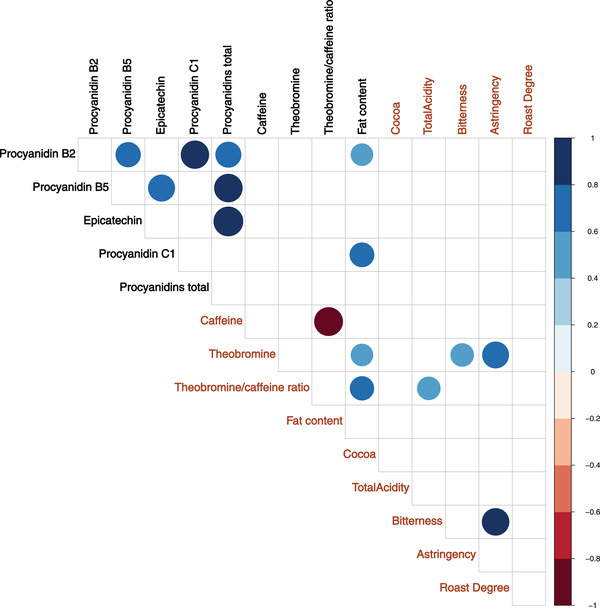
Correlation matrix of the results of near‐infrared spectroscopy determination of nonvolatile compounds (in unroasted beans) and sensory analysis (in liquors) belonging to the native Amazonian cocoa population. Nonvolatile compounds are shown in black and sensory traits are shown in bold and brown. The correlations were calculated by the Pearson method. The white boxes represent no significant correlations. The color of the circles corresponds to Pearson's correlation coefficient (R^2^ correlation coefficient). The scale on the right indicates the interpretations of different colors (blue for positive correlation and red for negative correlation). The size of the circles corresponds to the *p* value corresponding to the calculation of each correlation coefficient. The *p* value threshold for a significant correlation is .05

The NIRS analyses revealed nine different traits. Strong positive and negative correlations could be observed between the different traits measured by NIRS. As in the case of the modern Nacional population, the presence of one type of polyphenol is correlated with the presence of all the other detected polyphenols (Figure 3). No strong correlation could be observed between the different compounds identified by NIRS and the results of the sensory analyses (Figure 3).

The PCA results from the NIRS results also show a continuous variation within the population (Figure 2B). Axis 1 is predominantly influenced by the concentrations of total procyanidin, B2 procyanidin, and B5 procyanidin. Axis 2 is mainly influenced by the theobromine/caffeine ratio, caffeine content, and protein content.

### 2.3 Nacional population vs. native Amazonian cacao populations

**FIGURE 4:**
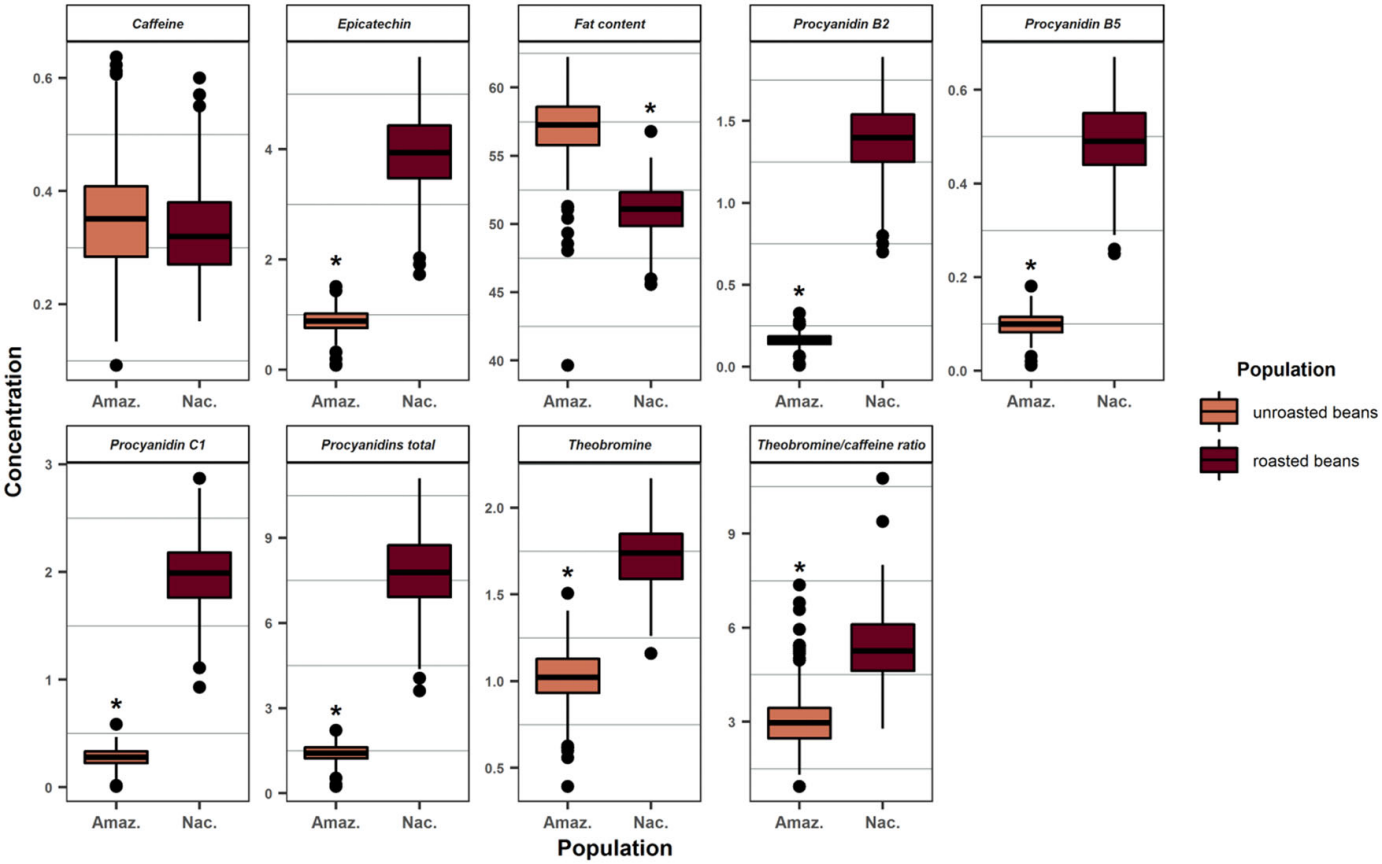
Boxplots representing the distribution of concentrations for each trait as a function of the cocoa tree population. A Student's t test was performed with a confidence level of 5%. Significantly different whisker boxes were annotated with a star. Unroasted beans from the Amazonian population (in orange) and roasted beans from the Nacional population (in brown). Amaz., Amazonian population; Nac., Nacional population

Significant differences are observed between the concentrations and their variations among the nine traits measured by NIRS depending on the cocoa tree population and the bean treatment (Figure 4). Cocoa beans from the modern Nacional population (roasted beans) thus appeared to be richer in epicatechin, procyanidin B2, procyanidin B5, procyanidin C1, total procyanidin, theobromine and had a higher theobromine/caffeine ratio (Figure 4). Cocoa beans from Amazonian population (unroasted beans) seem to have more fat content (Figure 4). As roasting is known in cocoa to lower the polyphenol content (Ioannone et al., 2015; Priftis et al., 2015), it seems that the Nacional population contains much more polyphenols than the Amazonian population.

**FIGURE 5:**
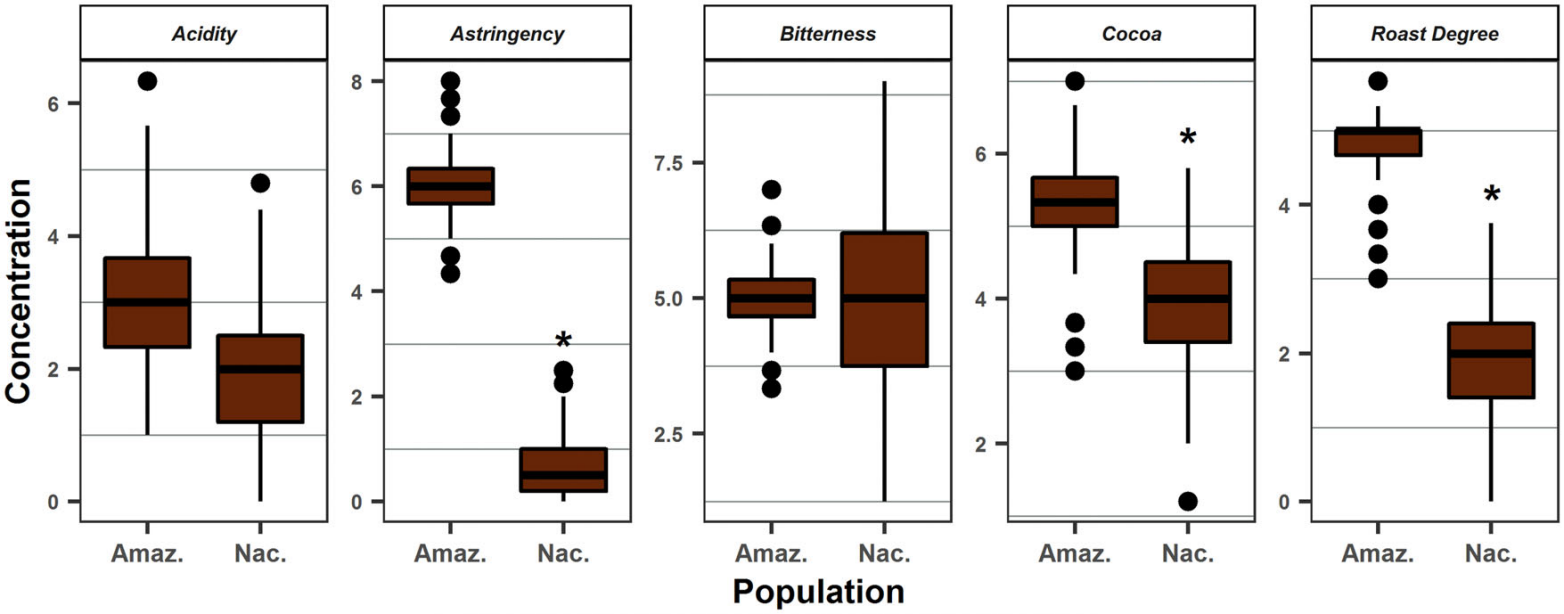
Boxplots representing the distribution of sensorial notes (made in liquors) for each trait as a function of the cocoa tree population. A Student's t test was performed with a confidence level of 5%. Significantly different whisker boxes were annotated with a star. Amaz., Amazonian population; Nac., Nacional population

The results of analyses of cocoa liquors from Amazonian trees and trees of the Nacional population, all made from roasted beans, show that Nacional population is less astringent with a less pronounced cocoa taste and a lower taste of degree of roast (Figure 5).

### 2.4 Identification of significant associations for biochemical compounds

All the significant association areas can be found in Supplemental Table S2.

Forty‐five areas of significant associations were detected in relation to the biochemical compounds evaluated by NIRS analyses (two in the modern Nacional population and 51 in the Amazonian population). All the association zones are shown in Supplemental Figure S1. The most important locus was detected for fat content on the chromosome 4 at the position 30,006,933 bp with a *p* value of 1.08 × 10^−11^.

#### 2.4.1 Identification of significant associations for biochemical compounds involved in the polyphenol biosynthetic pathway

**FIGURE 6:**
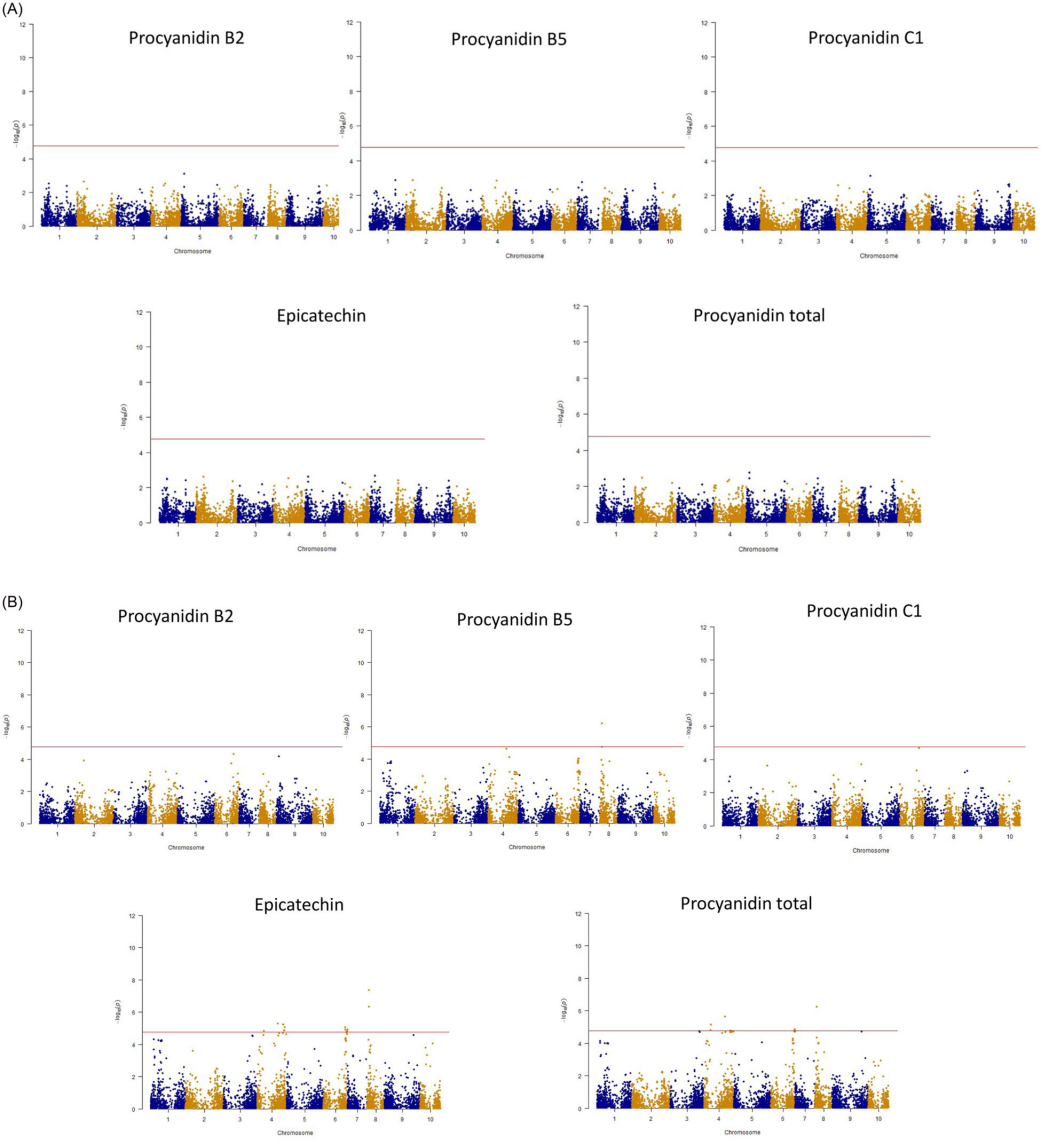
Manhattan plot representing the marker associations rate linked to polyphenols traits in cocoa beans. (A) Manhattan plot linked to polyphenols traits in Nacional population of cocoa trees. (B) Manhattan plot linked to polyphenols traits in the Amazonian population of cocoa trees. The red line represents the threshold of significant association

No association zones were detected for polyphenol content in the modern Nacional population (Figure 6A). Of the 45 association zones detected in the Amazonian population, 14 were detected in relation to the concentration of polyphenols, determined by NIRS in the population, on chromosomes 4, 6, and 8 (Figure 6B). Two colocations are present on chromosome 4 and one on chromosome 6 in relation to epicatechin and total procyanidin concentration. One colocation on chromosome 8 is present in relation to epicatechin, procyanidin B5, and total procyanidin concentration (Supplemental Table S2, Supplemental Figure S1). The most important loci detected for procyanidin B5, epicatechin and procyanidin total was detected at the same position on the chromosome 8 at the position 1,925,624 bp with a *p* values, respectively, of 6.05 × 10^−7^, 4.42 × 10^−8^, and 5.93 × 10^−7^.

#### 2.4.2 Identification of significant associations for biochemical compounds involved in the purine biosynthetic pathway

**FIGURE 7:**
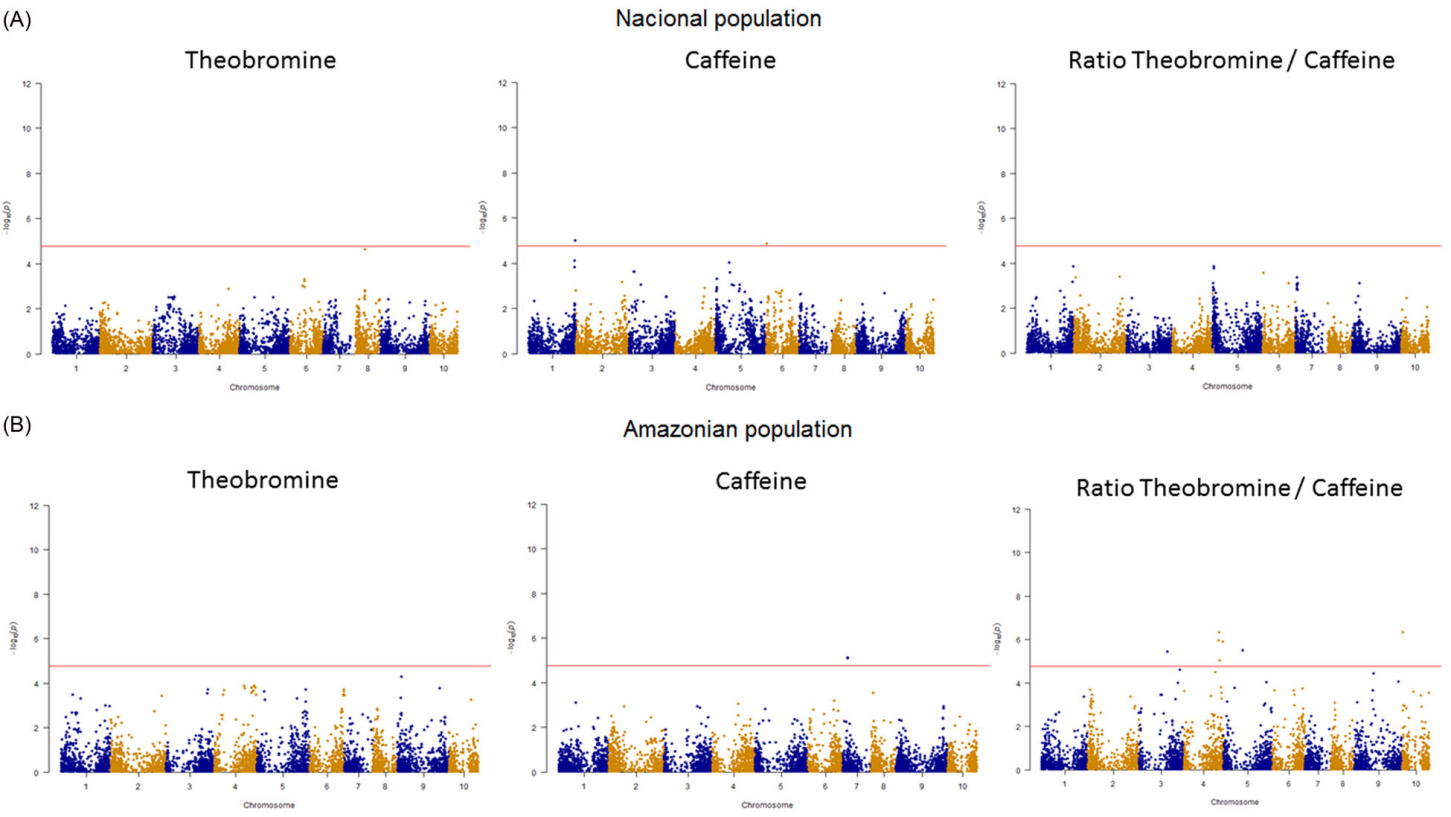
Manhattan plot representing the marker associations rate linked to caffeine and theobromine traits in cocoa beans. (A) Manhattan plot linked to caffeine and theobromine traits in Nacional population. (B) Manhattan plot linked to caffeine and theobromine traits in the Amazonian population. The red line represents the threshold of significant association

Of the two areas of association detected with the modern Nacional population, both were detected in relation to caffeine concentration on chromosomes 1 and 6 (Figure 7A).

Of the 45 areas of association detected with the Amazonian population, six were detected in relation to the concentration of caffeine or the theobromine/caffeine concentration ratio, determined by NIRS in the population, on chromosomes 3, 4, 5, 7, and 10 (Figure 7B).

No colocations between significant associations of the two populations were observed (Supplemental Table S2, Supplemental Figure S1).

The most important locus detected for the theobromine/caffeine ratio was detected on the chromosome 4 at the position 28,107,791 bp with a *p* value of 4.65 × 10^−7^. The most important locus detected for caffeine concentration was detected on the chromosome 1 at the position 36,651,730 bp with a *p* value of 2.96 × 10^−6^.

#### 2.4.3 Identification of significant associations for traits related to fat and protein content

**FIGURE 8:**
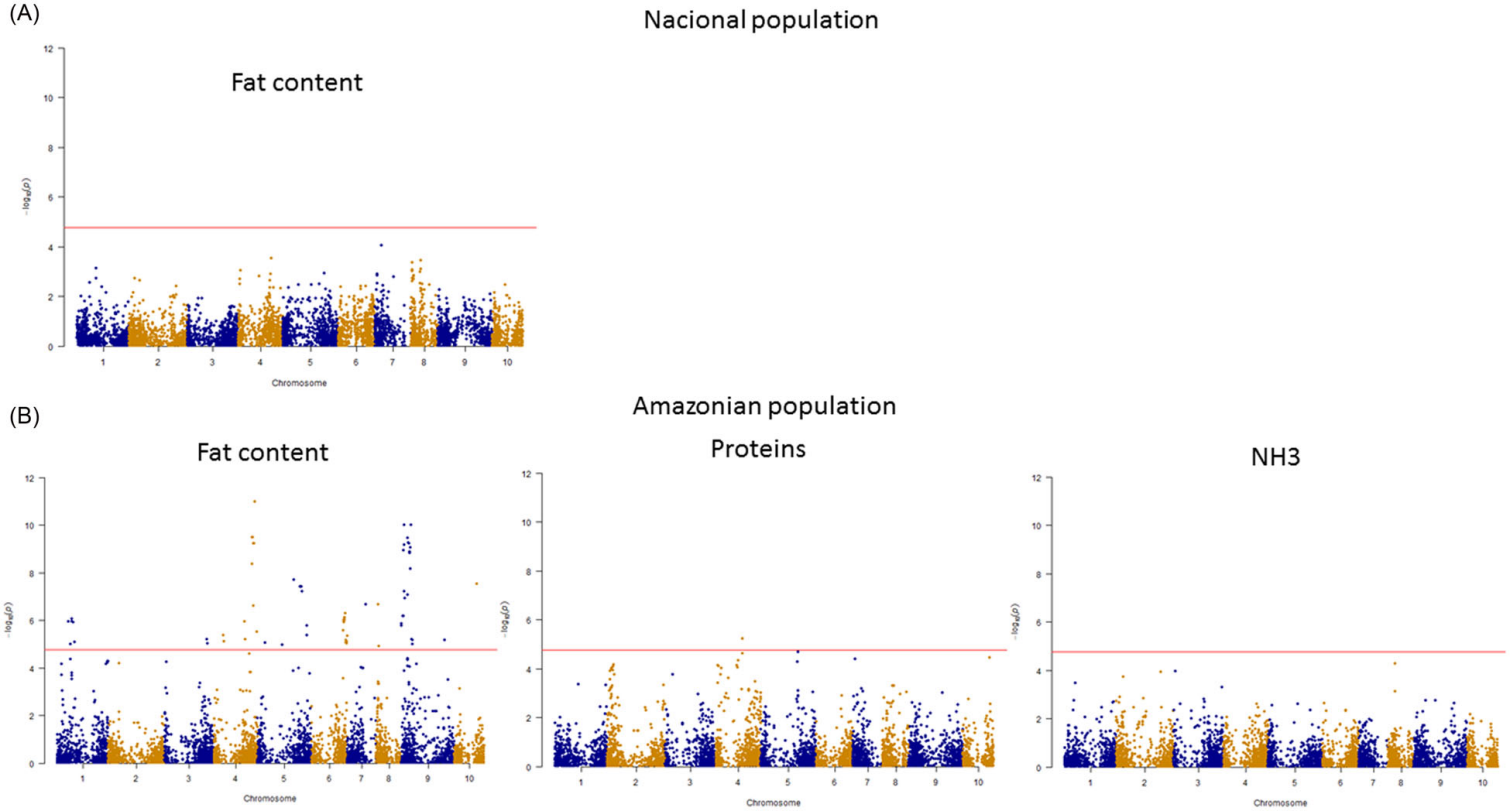
Manhattan plot representing the marker association's rate linked to fat and protein content traits in cocoa beans. (A) Manhattan plot linked to fat and protein content traits in Nacional population. (B) Manhattan plot linked to fat and protein content traits in the Amazonian population. The red line represents the threshold of significant association

No significant association was identified for the Nacional population (Figure 8A). Twenty‐two significant association areas were detected in relation to the fat content in the Amazonian population. They are located on all chromosomes except chromosome 2. One association zone was detected in relation to protein content located on chromosome 4 (Figure 8B).

No colocations between significant associations of the two populations were observed.

The most important locus detected for fat content was detected on the chromosome 4 at the position 30,006,933 bp with a *p* value of 1.08 × 10^−11^. The most important locusf detected for protein content was detected on the chromosome 4 at the position 18,831,023 bp with a *p* value of 6.02 × 10^−6^.

### 2.5 Identification of significant associations for sensory traits

Nineteen areas of association in relation to the scores established by the sensory analysis were detected (three in the modern Nacional population and 17 in the Amazonian population).

**FIGURE 9:**
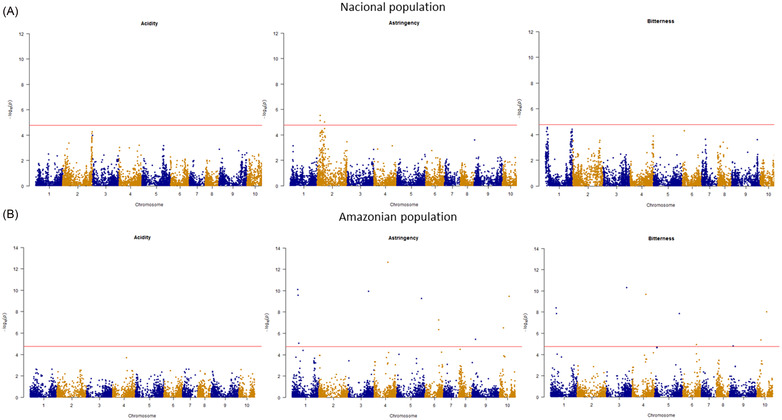
Manhattan plot representing the significant marker associations linked to astringency and bitterness traits in cocoa liquors. (A) Manhattan plot linked to astringency and bitterness traits in Nacional population. (B) Manhattan plot linked to astringency and bitterness traits in the Amazonian population. The red line represents the threshold of significant association

In the modern Nacional population, the three associations are related to astringency and are located on chromosome 2 (Figure 9A).

In the Amazonian population, 16 associations are related to bitterness and astringency (Supplemental Figure S1). The areas of interest were detected on chromosomes 1, 3, 4, 5, 6, 9, and 10 (Figure 9B; Supplemental Table S2). The perception of astringency and bitterness seems to be linked; eight colocations were detected between these two sensorial traits.

No colocations between the results of the two populations were observed.

The most important locus detected for astringency was detected on the chromosome 4 at the position 19,171,045 bp with a *p* value of 2.32 × 10^−13^. The most important locus detected for bitterness was detected on the chromosome 3 at the position 28,445,339 bp with a *p* value of 5.24 × 10^−11^.

### 2.6 Identification of candidate genes involved in the formation of biochemical compounds involved in bitterness

The set of association zones allowed the detection of 101 candidate genes potentially involved in the synthesis or degradation of the biochemical compounds identified by NIRS.

#### 2.6.1 Candidate genes potentially involved in the polyphenol biosynthetic pathway

**FIGURE 10:**
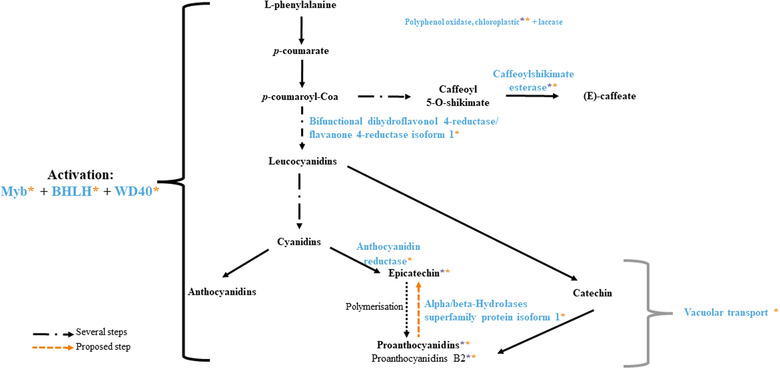
Diagram of the polyphenol biosynthetic pathway adapted from Chouhan et al. (2017) and Wollgast & Anklam (2000). Biochemical compounds are shown in bold. Candidate genes identified in the association zones are shown in blue in this diagram and arrows indicate their putative functions in the biosynthetic pathway. The purple stars show the compounds and candidate genes identified in the Nacional modern population cocoa trees. The orange stars show the compounds and candidate genes identified in the native Amazonian population cocoa trees. The black arrows show the biochemical modifications already identified in other publications. The orange dotted arrow shows the biochemical modifications proposed according to the results obtained

In the polyphenol association zones (epicatechin, procyanidin B5, and total procyanidin), 33 candidate genes were identified (Supplemental Table S3). Their putative action is shown in Supplemental Table S3. Their annotated functions in the genome indicate that these candidate genes would be involved in the biosynthetic pathway of polyphenols, particularly in the production of proanthocyanins, epicatechins, and catechol or in the activation of the biosynthetic pathway (Figure 10).

#### 2.6.2 Candidate genes potentially involved in the purine biosynthetic pathway

**FIGURE 11:**
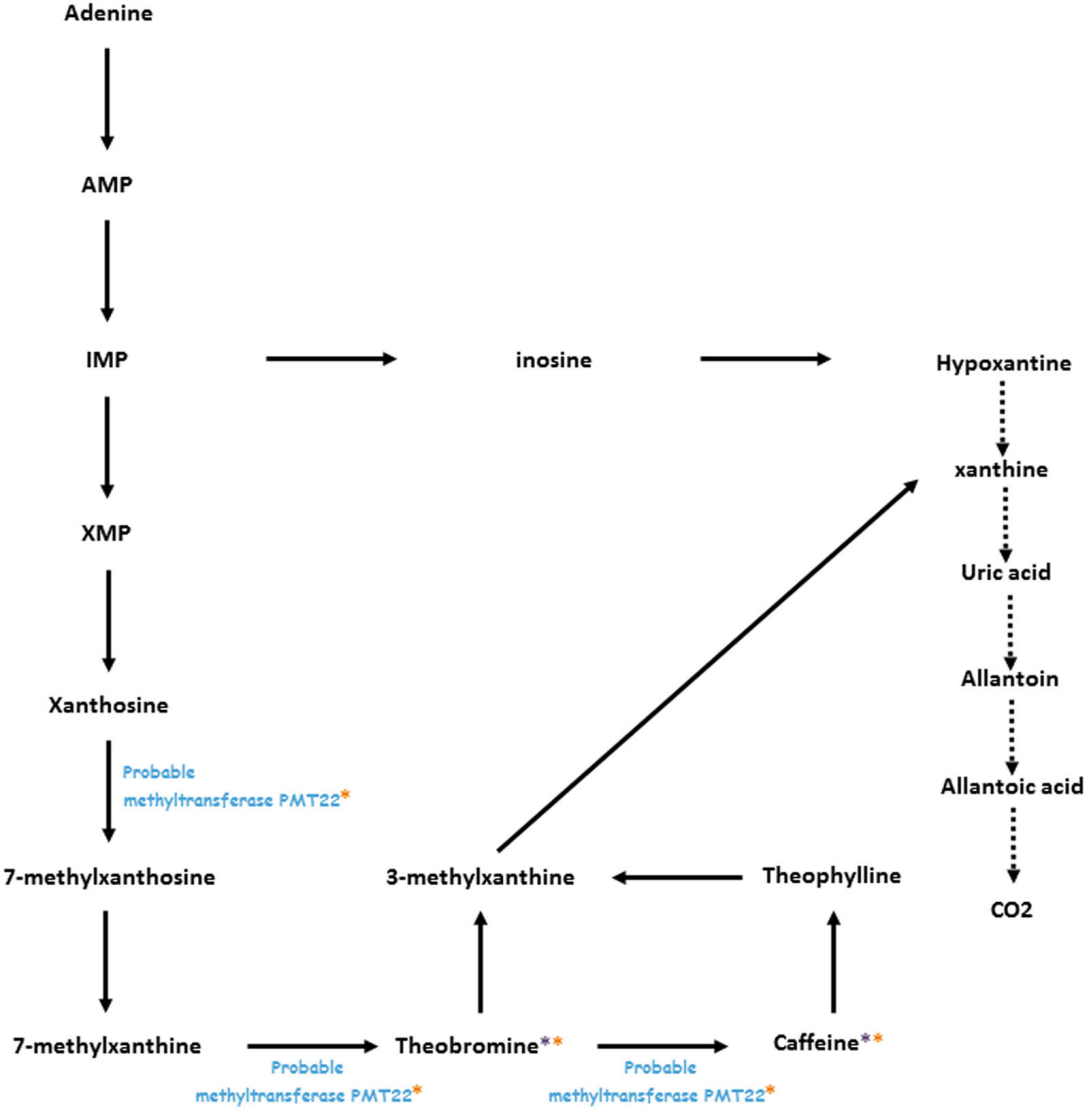
Scheme of caffeine biosynthesis, adapted from Zheng et al. (2004). Biochemical compounds are shown in bold. Candidate genes (in blue) located in the association zones are indicated at the side of arrows according to their putative functions in the biosynthetic pathway. The purple stars show the compounds and candidate genes identified in the Nacional modern population cocoa trees. The orange stars show the compounds and candidate genes identified in the native Amazonian population cocoa trees

One candidate gene has been identified in the purine compounds significant associations areas (Figure 11; Supplemental Table S3). This candidate genes could be involved three times in this biosynthetic pathway: in the production of 7‐methylxanthosine, theobromine, and caffeine.

#### 2.6.3 Candidate genes potentially involved in the fat biosynthetic and degradation pathway

Sixty‐two candidate genes were identified in the areas of association with fat content detected the Amazonian population. Of these candidate genes, 30 appear to be involved in the synthesis of fatty acids or their precursors, 29 in lipid catabolism, and five in the transport of fatty acids (Supplemental Table S3).

#### 2.6.4 Candidate genes involved in protein biosynthesis

Five candidate genes were identified in the significant association areas linked to protein content. Five of them have a function involved in protein transport and one gene has a hydrolase activity that could be responsible for the degradation of certain proteins (Supplemental Table S3).

## 3 DISCUSSION

Two different cocoa populations were analyzed in this work. Distinct results were observed between them. Sixty‐eight association zones linked to nonvolatile compounds and sensory analysis were detected for the Amazonian population and five for the Nacional population. Within these association zones, 81 candidate genes could be identified: one in purine biosynthesis; 64 in fatty acid synthesis, degradation, or transport; 10 in polyphenol biosynthesis; and six in protein biosynthesis.

Other quantitative trait loci studies have already been carried out using simple sequence repeat markers in relation to fat content but also to polyphenol content and the presence of bitterness and astringency revealed by sensory analyses (Araújo et al., 2009; Argout et al., 2011; Lanaud et al., 2003; Mustiga et al., 2019). Some of the results found in this study are common with previous studies. Three areas of association linked with fat content found in this new study colocate with the associations reported by Argout et al. (2011) on chromosomes 3, 7, 9. Three other areas of association with fat content found in this new study colocate with the associations found by Mustiga et al. (2019) on chromosomes 4, 5, and 9. One association zone linked with fat content found by Araújo et al. (2009) on chromosome 9 colocates with an association zone found in this study. One area of association linked with astringency found in this new study colocates with the association found by (Lanaud et al., 2003) on chromosome 1.

The Amazonian population showed more association areas (68) than the modern Nacional population (three) perhaps because of the larger genetic base of the Amazonian population (Supplemental Figure S3). This difference can have several causes. The two populations are genetically different. The Nacional population has a narrow genetic basis, explained by only three main highly homozygous ancestors, contrary to the Amazonian population, not selected, and which include native plants from Amazonia, with a higher allele richness (Loor Solorzano et al., 2015). Therefore, the allele diversity is reduced in the Nacional population, limiting the number of segregations and associations revealed.

It can be also partly explained by the different treatments that the beans underwent before the NIRS analyses. Indeed, the beans from the Nacional population were roasted in contrast to those from the Amazonian population. Roasting is known to have an impact on polyphenol content (Ioannone et al., 2015; Jinap et al., 1998; Misnawi et al., 2005; Priftis et al., 2015). In studies on cocoa and coffee roasting is responsible for the decrease in polyphenol content (Ioannone et al., 2015; Priftis et al., 2015), in others in coffee it is responsible for the increase in polyphenol content (Muzykiewicz‐Szymańska et al., 2021). Another study has shown that roasting protocol can also influence the capacity of polyphenols to interact with protein and decrease the potential of astringency (Misnawi et al., 2005). These observations could also explain why the genetic component is more difficult to detect for the Nacional population.

It can be concluded that cocoa from trees belonging to the Nacional cultivars (Nacional population) give beans with less astringency and a less strong cacao flavor. No significant differences were observed for acidity and bitterness. However, the Amazonian population tends to have a higher acidity than the Nacional population. The difference in bitterness between the two populations has not been demonstrated, but the Amazonian population shows a medium bitterness with little variation. The Nacional population, involving ancestors contrasted for this trait (Amelonado, Criollo, and Nacional) could explain its larger variability for this trait.

Only one candidate gene involved in the biosynthetic pathway of purine biosynthesis or protein biosynthesis has been identified. Further annotation of the cocoa genome could allow the identification of new genes. Furthermore, our method of searching for candidate genes based on annotations can be complemented with other methods without preconceptions to find genes whose function is not necessarily known.

On the side of its interaction with aroma, nonvolatile cocoa compounds, such as polyphenols, are also useful compounds for human health (Andújar et al., 2012; Cooper et al., 2008). Characteristics related to bitterness and astringency are important to consider when selecting clones to create new cultivars depending on the breeding objectives.

The results of our study have shown the polygenic nature of some traits as caffeine and theobromine content, fat content, and polyphenol content. These results could provide useful information to define breeding strategies adapted to these traits as a genomic selection strategy adapted to highly polygenic traits.

## AUTHOR CONTRIBUTIONS

Kelly Colonges: Data curation; Formal analysis; Investigation; Methodology; Validation; Visualization; Writing – original draft. Edward Seguine: Formal analysis. Alejandra Saltos: Formal analysis. Fabrice Davrieux: Formal analysis; Supervision. Jérôme Minier: Formal analysis. Juan‐Carlos Jimenez: Formal analysis. Marie‐Christine Lahon: Formal analysis. Darío Calderon: Formal analysis. Cristian Subia: Formal analysis. Ignacio Sotomayor: Formal analysis. Fabián Fernández: Formal analysis. Olivier Fouet: Data curation. Bénédicte Rhoné: Data curation. Xavier Argout: Data curation. Marc Lebrun: Formal analysis. Pierre Costet: Data curation; Funding acquisition. Claire Lanaud: Conceptualization; Funding acquisition; Investigation; Project administration; Supervision; Validation; Writing – review & editing. Renaud Boulanger: Conceptualization; Funding acquisition; Investigation; Project administration; Supervision; Validation; Writing – review & editing. Rey Gastón Loor Solorzano: Conceptualization.

## CONFLICT OF INTEREST

The authors declare that the research was conducted in the absence of any commercial or financial relationships that could be construed as a potential conflict of interest.

## Supporting information

Supplemental Figure S1: Graphical representation of LD decay. The blue curve represents the trend line of the linkage disequilibrium. The red vertical line represents the DL threshold.

Supplemental Figure S2: Physical map of the association zones. The peaks of the associations are represented by a circle whose diameter is proportional to the p‐value of the association. The rectangle on either side of the circle represents the confidence interval of the association zone. The areas of association detected with the modern Nacional population are represented in purple, those detected with the native Amazonian cocoa population are represented in green.

Supplemental Figure S3: Phylogenetic tree of both population, Amazonian and Nacional, of cocoa trees. In green are represented the studied individuals belonging to the Amazonian population and in purple the individuals of the Nacional population. The other individuals represented are the control individuals of the 10 known genetic groups in the cocoa tree. The name of each control genetic group is framed: Curaray, Nacional (corresponds to the ancestral Nacional), Nanay, Amelonado, Maranon, Guiana, Caqueta, Iquitos, Criollo, Purus.

Supplemental Table S1: List of accession used for analysis In the first column are listed the accessions used. In the second column, the type of analysis performed with this accession is listed. In the third column the type of population is indicated (Nacional = Nacional population; Amazonian: native Amazonian cocoa population).

Supplemental Table S2: Complete list of association zones.

Supplemental Table S3: Complete list of candidate genes. When no candidate gene was found, genes close to the areas of association were studied. Genes found outside the confidence intervals are noted as “_near”.
