# Supplementary material for: Diversity and determinants of bitterness, astringency, and fat content in cultivated Nacional and native Amazonian cocoa accessions from Ecuador
Source: Plant Genome. 2022 Sep 6;15(4):e20218. doi: 10.1002/tpg2.20218 (PMC12806967; doi:10.1002/tpg2.20218)
Supplement: Supplementary file 1 [file TPG2-15-e20218-s001.pdf]

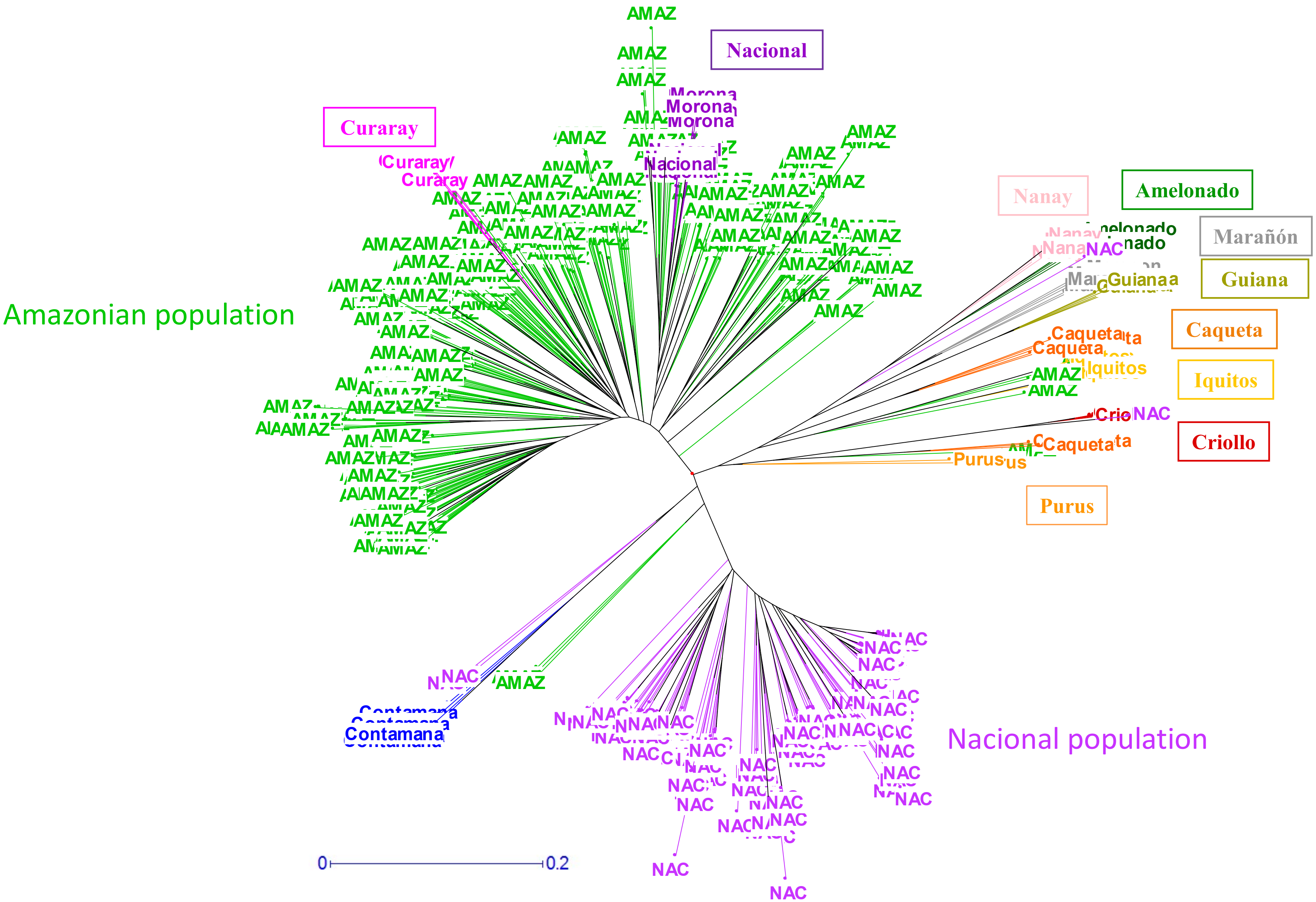
Nacional
Curaray
Amazonian population
Nanay
Amelonado
Marañón
Guiana
Caqueta
Iquitos
Criollo
Purus
Contamana
Nacional population
Morona
AMAZ
NAC
0
0.2
