# Supplementary material for: Diversity and determinants of bitterness, astringency, and fat content in cultivated Nacional and native Amazonian cocoa accessions from Ecuador
Source: Plant Genome. 2022 Sep 6;15(4):e20218. doi: 10.1002/tpg2.20218 (PMC12806967; doi:10.1002/tpg2.20218)
Supplement: Supplementary file 2 [file TPG2-15-e20218-s004.pdf]

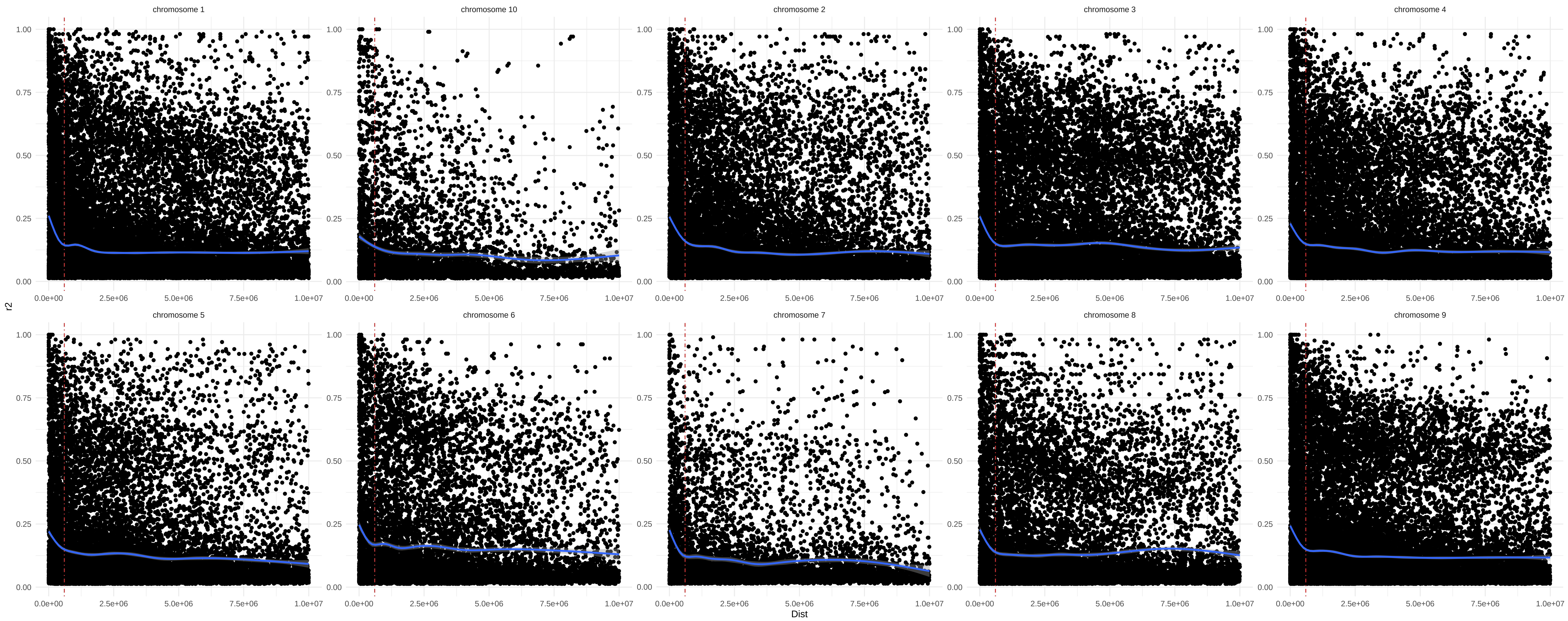

chromosome 1
chromosome 10
chromosome 2
chromosome 3
chromosome 4
chromosome 5
chromosome 6
chromosome 7
chromosome 8
chromosome 9
r2
Dist
0.00
0.25
0.50
0.75
1.00
0.0e+00
2.5e+06
5.0e+06
7.5e+06
1.0e+07
