# Supplementary material for: Diversity and determinants of bitterness, astringency, and fat content in cultivated Nacional and native Amazonian cocoa accessions from Ecuador
Source: Plant Genome. 2022 Sep 6;15(4):e20218. doi: 10.1002/tpg2.20218 (PMC12806967; doi:10.1002/tpg2.20218)
Supplement: Supplementary file 3 [file TPG2-15-e20218-s006.pdf]

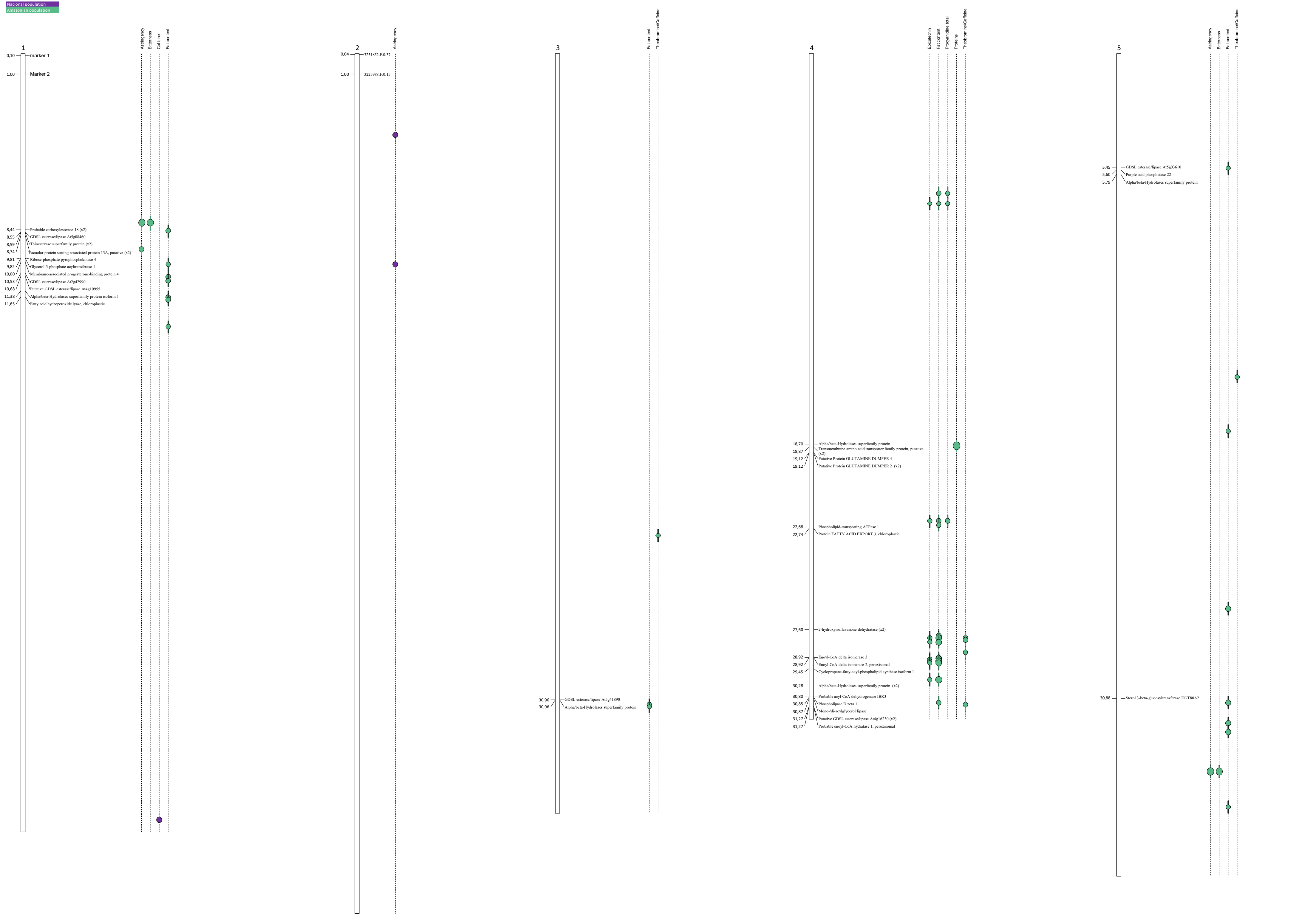

Nacional population
Amazonian population
1
Astringency
Bitterness
Caffeine
Fat content
0,10 marker 1
1,00 Marker 2
8,44 Probable carboxylesterase 18 (x2)
8,55 GDSL esterase/lipase At5g08460
8,59 Thioesterase superfamily protein (x2)
8,74 Vacuolar protein sorting-associated protein 13A, putative (x2)
9,81 Ribose-phosphate pyrophosphokinase 4
9,82 Glycerol-3-phosphate acyltransferase 1
10,00 Membrane-associated progesterone-binding protein 4
10,53 GDSL esterase/lipase At2g42990
10,68 Putative GDSL esterase/lipase At4g10955
11,38 Alpha/beta-Hydrolases superfamily protein isoform 1
11,65 Fatty acid hydroperoxide lyase, chloroplastic
2
Astringency
0,04 3251852.F.0.37
1,00 3225988.F.0.15
3
Fat content
Theobromine/Caffeine
30,96 GDSL esterase/lipase At5g41890
30,96 Alpha/beta-Hydrolases superfamily protein
4
Epicatechin
Fat content
Procyanidine total
Proteins
Theobromine/Caffeine
18,70 Alpha/beta-Hydrolases superfamily protein
18,87 Transmembrane amino acid transporter family protein, putative (x2)
19,12 Putative Protein GLUTAMINE DUMPER 4
19,12 Putative Protein GLUTAMINE DUMPER 2 (x2)
22,68 Phospholipid-transporting ATPase 1
22,74 Protein FATTY ACID EXPORT 3, chloroplastic
27,60 2-hydroxyisoflavanone dehydratase (x2)
28,92 Enoyl-CoA delta isomerase 3
28,92 Enoyl-CoA delta isomerase 2, peroxisomal
29,45 Cyclopropane-fatty-acyl-phospholipid synthase isoform 1
30,28 Alpha/beta-Hydrolases superfamily protein (x2)
30,80 Probable acyl-CoA dehydrogenase IBR3
30,85 Phospholipase D zeta 1
30,87 Mono-/di-acylglycerol lipase
31,27 Putative GDSL esterase/lipase At4g16230 (x2)
31,27 Probable enoyl-CoA hydratase 1, peroxisomal
5
Astringency
Bitterness
Fat content
Theobromine/Caffeine
5,45 GDSL esterase/lipase At5g03610
5,60 Purple acid phosphatase 22
5,79 Alpha/beta-Hydrolases superfamily protein
30,88 Sterol 3-beta-glucosyltransferase UGT80A2

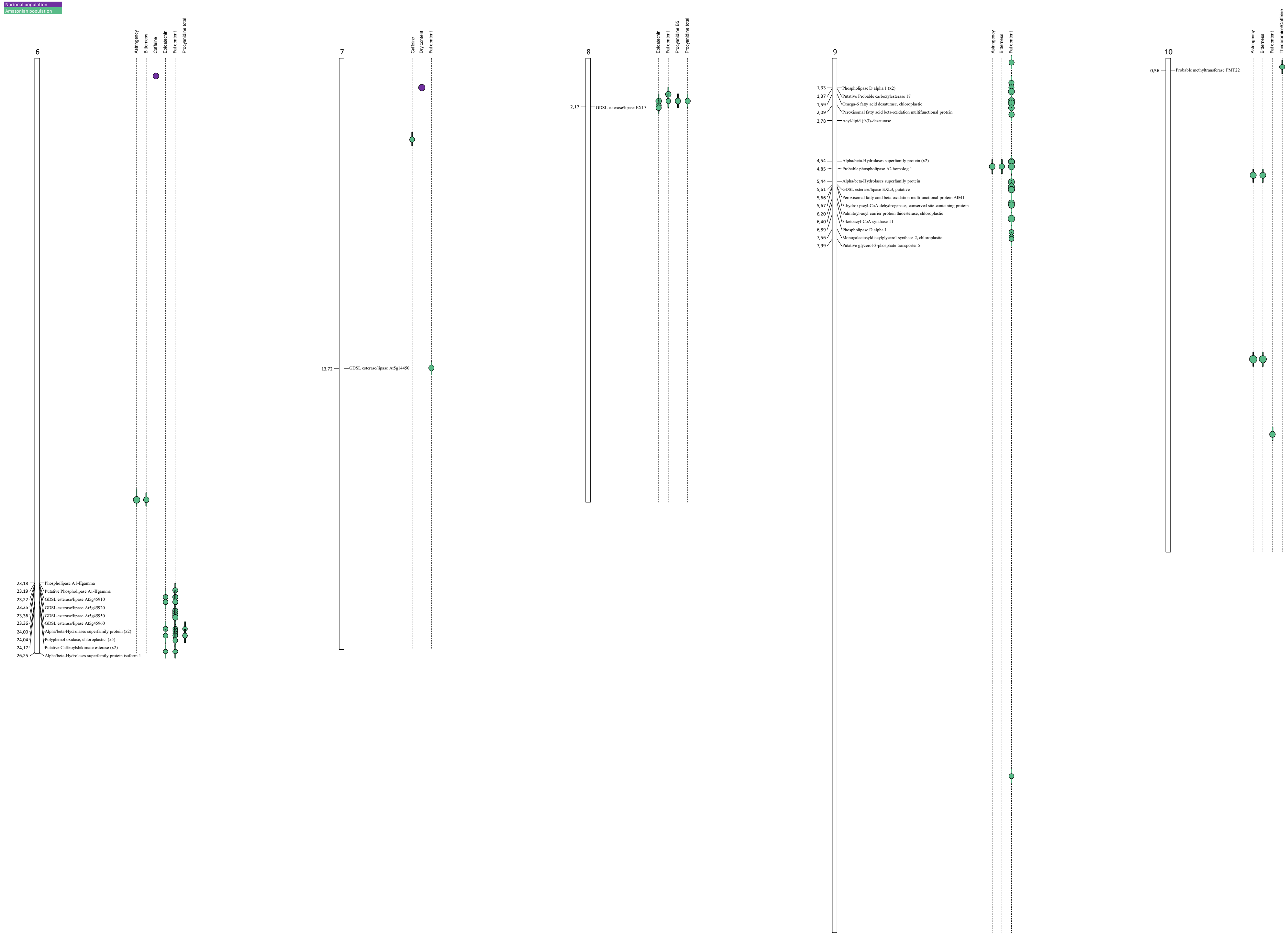

Nacional population
Amazonian population
6
Astringency
Bitterness
Caffeine
Epicatechin
Fat content
Procyanidine total
23,18 Phospholipase A1-IIgamma
23,19 Putative Phospholipase A1-IIgamma
23,22 GDSL esterase/lipase At5g45910
23,25 GDSL esterase/lipase At5g45920
23,36 GDSL esterase/lipase At5g45950
23,36 GDSL esterase/lipase At5g45960
24,00 Alpha/beta-Hydrolases superfamily protein (x2)
24,04 Polyphenol oxidase, chloroplastic (x5)
24,17 Putative Caffeoylshikimate esterase (x2)
26,25 Alpha/beta-Hydrolases superfamily protein isoform 1
7
Caffeine
Dry content
Fat content
13,72 GDSL esterase/lipase At5g14450
8
Epicatechin
Fat content
Procyanidine B5
Procyanidine total
2,17 GDSL esterase/lipase EXL3
9
Astringency
Bitterness
Fat content
1,33 Phospholipase D alpha 1 (x2)
1,37 Putative Probable carboxylesterase 17
1,59 Omega-6 fatty acid desaturase, chloroplastic
2,09 Peroxisomal fatty acid beta-oxidation multifunctional protein
2,78 Acyl-lipid (9-3)-desaturase
4,54 Alpha/beta-Hydrolases superfamily protein (x2)
4,85 Probable phospholipase A2 homolog 1
5,44 Alpha/beta-Hydrolases superfamily protein
5,61 GDSL esterase/lipase EXL3, putative
5,66 Peroxisomal fatty acid beta-oxidation multifunctional protein AIM1
5,67 3-hydroxyacyl-CoA dehydrogenase, conserved site-containing protein
6,20 Palmitoyl-acyl carrier protein thioesterase, chloroplastic
6,40 3-ketoacyl-CoA synthase 11
6,89 Phospholipase D alpha 1
7,56 Monogalactosyldiacylglycerol synthase 2, chloroplastic
7,99 Putative glycerol-3-phosphate transporter 5
10
Astringency
Bitterness
Fat content
Theobromine/Caffeine
0,56 Probable methyltransferase PMT22
